# Prognostic Role of Mismatch Repair Status, Histotype and High-Risk Pathologic Features in Stage II Small Bowel Adenocarcinomas

**DOI:** 10.1245/s10434-020-08926-4

**Published:** 2020-08-05

**Authors:** Alessandro Vanoli, Federica Grillo, Camilla Guerini, Giuseppe Neri, Giovanni Arpa, Catherine Klersy, Gabriella Nesi, Paolo Giuffrida, Gianluca Sampietro, Sandro Ardizzone, Paolo Fociani, Roberto Fiocca, Giovanni Latella, Fausto Sessa, Antonietta D’Errico, Deborah Malvi, Claudia Mescoli, Massimo Rugge, Stefano Ferrero, Gilberto Poggioli, Fernando Rizzello, Maria C. Macciomei, Donatella Santini, Umberto Volta, Roberto De Giorgio, Giacomo Caio, Antonio Calabrò, Carolina Ciacci, Maria D’Armiento, Aroldo Rizzo, Gaspare Solina, Michele Martino, Francesco Tonelli, Vincenzo Villanacci, Renato Cannizzaro, Vincenzo Canzonieri, Ada Maria Florena, Livia Biancone, Giovanni Monteleone, Roberto Caronna, Antonio Ciardi, Luca Elli, Flavio Caprioli, Maurizio Vecchi, Renata D’Incà, Fabiana Zingone, Anna D’Odorico, Marco Vincenzo Lenti, Barbara Oreggia, Luca Reggiani Bonetti, Antonino Giulio Giannone, Augusto Orlandi, Valeria Barresi, Rachele Ciccocioppo, Giuseppe Amodeo, Elena Biletta, Ombretta Luinetti, Paolo Pedrazzoli, Andrea Pietrabissa, Gino Roberto Corazza, Enrico Solcia, Marco Paulli, Antonio Di Sabatino

**Affiliations:** 1 Anatomic Pathology Unit, Department of Molecular Medicine, University of Pavia and Fondazione IRCCS San Matteo Hospital, Pavia, Italy; 2 Pathology Unit, Department of Surgical and Diagnostic Sciences, University of Genoa and Ospedale Policlinico San Martino University Hospital, Genoa, Italy; 3 Clinical Epidemiology and Biometry Unit, Fondazione IRCCS San Matteo Hospital, Pavia, Italy; 4 Division of Pathological Anatomy, Department of Surgery and Translational Medicine, University of Florence, Florence, Italy; 5 Department of Internal Medicine, University of Pavia and Fondazione IRCCS San Matteo Hospital, Pavia, Italy; 6 Unit of General Surgery, ASST Rhodense, Rho Hospital, University of Milan, Milan, Italy; 7 Gastroenterology Unit, Luigi Sacco University Hospital, Milan, Italy; 8 Anatomic Pathology Unit, ASST Ovest Milanese, Milan, Italy; 9 Gastroenterology Unit, Department of Life and Environmental Sciences, University of L’Aquila, L’Aquila, Italy; 10 Pathology Unit, Department of Medicine and Surgery, University of Insubria, Varese, Italy; 11 Department of Experimental, Diagnostic and Specialty Medicine (DIMES), Institute of Oncology and Transplant Pathology, St. Orsola-Malpighi Hospital, University of Bologna, Bologna, Italy; 12 Pathology Unit, Department of Medicine, University of Padua, Padua, Italy; 13 Division of Pathology, Fondazione IRCCS Ca’ Granda Ospedale Maggiore Policlinico, Department of Biomedical, Surgical and Dental Sciences, University of Milan, Milan, Italy; 14 Surgery of the Alimentary Tract, Department of Medical and Surgical Sciences, Sant’Orsola—Malpighi Hospital, University of Bologna, Bologna, Italy; 15 Intestinal Chronic Bowel Disease Unit, Department of Medical and Surgical Sciences, Sant’Orsola—Malpighi Hospital, Alma Mater Studiorum University of Bologna, Bologna, Italy; 16 Pathology Unit, San Camillo-Forlanini Hospital, Rome, Italy; 17 Department of Medical and Surgical Sciences, University of Bologna, Bologna, Italy; 18 Department of Morphology, Surgery and Experimental Medicine, University of Ferrara, Ferrara, Italy; 19 Department of Experimental and Clinical Biomedical Sciences, University of Florence, Florence, Italy; 20 Department of Medicine and Surgery, University of Salerno, Salerno, Italy; 21 Public Health Department, Federico II University of Naples, Naples, Italy; 22 Unit of Pathology, Cervello Hospital, Palermo, Italy; 23 Units of General Surgery, Cervello Hospital, Palermo, Italy; 24 Surgery and Translational Medicine, University of Florence, Florence, Italy; 25 Institute of Pathology, Spedali Civili Hospital, Brescia, Italy; 26 Department of Gastroenterology, Centro di Riferimento Oncologico (CRO) di Aviano IRCCS, Aviano, Italy; 27 Pathology Unit, Centro di Riferimento Oncologico (CRO) di Aviano IRCCS, Aviano, Italy; 28 Department of Medical, Surgical and Health Sciences, University of Trieste, Trieste, Italy; 29 Pathologic Anatomy Unit, Department of Health Promotion, Mother and Child Care, Internal Medicine and Medical Specialties, University of Palermo, Palermo, Italy; 30 Department of Systems Medicine, University of Tor Vergata, Rome, Italy; 31 Department of Surgical Sciences, La Sapienza University, Rome, Italy; 32 Department of Radiological, Oncological, Pathological Sciences, Umberto I Hospital, La Sapienza University, Rome, Italy; 33 Gastroenterology and Endoscopy Unit, Fondazione IRCCS Ca’ Granda Ospedale Maggiore Policlinico, Milan, Italy; 34 Gastroenterology Section, Department of Surgery, Oncology and Gastroenterology, University of Padua, Padua, Italy; 35 General Surgery Unit, Ca’ Granda-Ospedale Maggiore Policlinico, Milan, Italy; 36 Section of Pathology, Department of Diagnostic Medicine and Public Health, University of Modena and Reggio Emilia, Modena, Italy; 37 Department of Biopathology and Image Diagnostics, University of Tor Vergata, Rome, Italy; 38 Section of Anatomical Pathology, Department of Diagnostics and Public Health, University and Hospital Trust of Verona, Verona, Italy; 39 Gastroenterology Unit, Department of Medicine, AOUI Policlinico G.B. Rossi, University of Verona, Verona, Italy; 40 Anatomic Pathology ASL Biella, Biella, Italy; 41 Oncology Unit, IRCCS San Matteo Hospital, Pavia, Italy; 42 Department of Surgery, General Surgery II, University of Pavia and Fondazione IRCCS San Matteo Hospital, Pavia, Italy

## Abstract

**Background:**

Small bowel adenocarcinoma is a relatively rare cancer, often diagnosed in an advanced stage. In localized and resectable disease, surgery alone or in combination with adjuvant chemotherapy is the mainstay of treatment. In the recently published National Comprehensive Cancer Network Clinical Practice guidelines, criteria for selecting patients with stage II small bowel adenocarcinoma to receive adjuvant chemotherapy are provided, and they are mainly extrapolated from studies on colorectal cancer.

**Patients and Methods:**

In the present study, we aimed to verify whether mismatch repair deficiency phenotype, high-risk pathologic features (including T4, positive resection margins and a low number of lymph nodes harvested), as well as tumor histologic subtype, were associated with cancer-specific survival in 66 stage II non-ampullary small bowel adenocarcinoma patients, collected through the Small Bowel Cancer Italian Consortium. A central histopathology review was performed. Mismatch repair deficiency was tested by immunohistochemistry for MLH1, MSH2, MSH6 and PMS2, and confirmed by polymerase chain reaction for microsatellite instability.

**Results:**

We identified mismatch repair deficiency, glandular/medullary histologic subtype, and celiac disease as significant predictors of favorable cancer-specific survival using univariable analysis with retained significance in bivariable models adjusted for pT stage. Among the high-risk features, only T4 showed a significant association with an increased risk of death; however, its prognostic value was not independent of mismatch repair status.

**Conclusions:**

Mismatch repair protein expression, histologic subtype, association with celiac disease, and, in the mismatch repair proficient subset only, T stage, may help identify patients who may benefit from adjuvant chemotherapy.

**Graphic Abstract:**

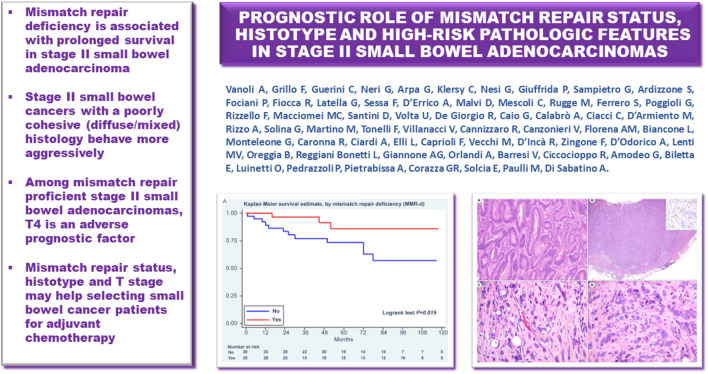

Small bowel adenocarcinomas (SBAs) are relatively rare tumors and account for 30–40% of all small intestine malignancies.^1^^,^^2^ They are often diagnosed at locally advanced or metastatic stages, which are associated with poor patient prognosis due to limited therapeutic options.^3^ In localized and resectable disease, surgery alone or in combination with adjuvant chemotherapy (ACT) represents the mainstay of treatment. However, the clinical benefit of ACT in SBAs is a matter of debate, especially for stage II tumors, which represent about 45% of resected SBA series.^4^ Stage II SBAs show a 5-year cancer-specific survival of only 55%, much lower than that of stage II colorectal cancer patients, which is reported to be 84%.^5^ French intergroup guidelines stated that ACT may be discussed for stage II patients with pT4 tumors (expert agreement).^6^ In the recently published National Comprehensive Cancer Network (NCCN) Clinical Practice guidelines, Small Bowel Adenocarcinoma, Version 1.2020, the criteria for selecting patients with stage II SBAs for ACT are mainly extrapolated from colorectal cancer studies and include: (1) mismatch repair (MMR) or microsatellite instability (MSI) status; and (2) presence of high-risk pathologic features, namely low number of isolated lymph nodes, pT4/tumor perforation, and positive resection margins.^7^ Additional factors which may be considered are lympho-vascular invasion, perineural invasion and high histologic grade^7^.

MMR deficiency (MMR-d) has been reported in up to 30–40% of resected SBAs and it has been found to be associated with etiology, being typical of Lynch syndrome-associated SBAs and frequent in celiac disease-associated cancers.^8^^–^13 The prognostic significance of MMR-d in resected SBAs has been investigated in several studies, with a favorable impact of MMR-d using univariable analyses in most studies, despite non-uniform results using stage-inclusive multivariate analyses.^8^^,^^10^, ^12^^–^16 Although MMR-d has been definitively associated with a better survival in stage II colorectal cancers, studies specifically addressing the same issue in stage II SBAs are lacking.

The aim of our study was to verify whether MMR-d phenotype, high-risk pathologic features endorsed by NCCN guidelines, as well as tumor histologic subtype, are associated with cancer-specific survival in a relatively large and well-characterized series of stage II SBAs collected through the Small Bowel Cancer Italian Consortium.

## Materials and Methods

### Study Population

This retrospective study included patients with primary, non-ampullary, resected stage II SBAs, retrieved from a larger population of 149 SBAs, enrolled from 22 tertiary referral Italian Centers participating in the Small Bowel Cancer Italian Consortium. Demographic features, tumor site, and the presence of a hereditary or immune-mediated predisposing condition were recorded. Diagnosis of celiac disease was based on serum IgA anti-endomysial and anti-tissue transglutaminase antibody positivity associated with typical duodenal histopathological lesions.^17^ Diagnosis of Crohn’s disease was ascertained according to international criteria;^18^ the site and extent of the disease were confirmed by endoscopy, histology and imaging. Lynch syndrome was defined by the presence of MMR deficiency due to constitutional pathogenic mutations affecting an MMR gene (MLH1, MSH2, MSH6, or PMS2).^19^ This study was approved by the Ethics Committee of the IRCCS (Istituto di Ricovero e Cura a Carattere Scientifico) San Matteo Hospital Foundation in Pavia (protocol number 20140003980).

### Histology, Immunohistochemistry and Molecular Analyses

Tissue samples were fixed in 4% formaldehyde and embedded in paraffin wax. All cases were investigated for histologic subtype and for all the parameters required to fulfil the criteria of the eighth edition of the American Joint Committee on Cancer (AJCC) TNM staging system.^3^ Histologically, small bowel carcinomas were classified as glandular (conventional adenocarcinomas), diffuse/poorly cohesive (exhibiting diffusely infiltrating and poorly cohesive cells, with little to no gland formation in more than 70% of the tumor), mixed (showing a combination of both glandular and poorly cohesive cell patterns, constituting at least 30% each), or medullary-type (characterized by a prominent T lymphoid infiltration and a pushing margin), as previously reported.^20^ Glandular and medullary cancers were grouped together to form a cohesive histologic subtype, and diffuse and mixed cancers were grouped together to form a non-cohesive subtype. For immunohistochemistry, 4-μm-thick sections were stained on a Dako Omnis platform with the following antibodies: MLH1 (monoclonal, clone ES05, prediluted, Dako), MSH2 (monoclonal, clone FE11, prediluted, Dako), MSH6 (monoclonal, clone EP49, prediluted, Dako), and PMS2 (monoclonal, clone EP51, prediluted, Dako). Immunostaining of MMR proteins in tumor cells was evaluated as proficient (MMR-p), if nuclear expression was retained, or deficient (MMR-d) if nuclear staining was absent, in the presence of an internal positive control, represented by intra-tumor stromal or inflammatory cells or non-tumor mucosa. In parallel, MSI molecular testing was performed, as previously reported.^12^*MLH1* methylation status was examined by pyrosequencing in SBAs showing loss of MLH1 immunohistochemical expression, as previously described.^12^

### Evaluation of High-Risk and Extended High-Risk Features

The presence or absence of all high-risk features endorsed by NCCN guidelines, including pT4, positive surgical margins, and a low number of lymph nodes, were recorded.^7^ In addition, extended high-risk features incorporating lymphovascular/perineural invasion and high histologic grade were also assessed.^7^^,^^21^^–^24 Surgical resection margins were classified as R0 (negative) or R1 (microscopically positive). The number of examined lymph nodes was regarded as low (when fewer than five lymph nodes for duodenal and fewer than eight for jejunal/ileal neoplasms were retrieved) or as adequate (≥ 5 lymph nodes for duodenal and ≥ 8 lymph nodes for jejunal/ileal neoplasms). Lymphovascular or perineural invasion was searched for in representative hematoxylin and eosin-stained tumor sections. In cases without unequivocal evidence of lymphovascular invasion on hematoxylin and eosin-stained sections, immunohistochemistry for the endothelial marker CD31 (monoclonal, clone JC70A, Dako) was also performed to improve detection. Histologic grade was categorized as high (G3 or poorly differentiated tumors), when < 50% of the tumor was composed of glands, or low (well-to-moderately differentiated tumors, G1–G2), when ≥ 50% of tumor was composed of glands.

All parameters were determined by reviewing both histologic slides and pathology reports. A central pathology review of each case was performed by at least two gastrointestinal pathologists (AV and ES).

### Statistical Analysis

Stata 16.1 (StataCorp, College Station, TX, USA) was used for all analyses. A two-sided *P* value < 0.05 was considered statistically significant. The data were described with the mean and standard deviation (SD) if continuous and with counts and percentages if categorical; they were compared between groups with the Student *t* test or the Fisher test, respectively. Variables with a *P* < 0.1 on univariable analysis were included in a multivariate exact logistic model. Median follow-up (25–75th percentile) was computed with the reverse Kaplan–Meier method. Follow-up was computed from diagnosis of cancer to death or last available follow-up for censored patients. Cumulative survival curves were plotted according to the Kaplan–Meier method and compared with the log-rank test. The strength of the association between series of candidate risk factors and cancer-specific mortality was assessed using Cox regression; hazard ratios and 95% CI were derived from the models. Owing to the limited number of events, only bivariable models were fitted to adjust, in turn, for MMR-d, celiac disease, histologic subtype, and pT stage.

## Results

This retrospective study included a cohort of 66 patients with pathologically confirmed primary extra-ampullary stage II resected SBAs. Demographic and clinicopathologic data of all patients evaluated are reported in Table 1. We recruited 21 patients with celiac disease associated-SBA, 20 with Crohn’s disease associated-SBA, 18 sporadic, and 7 cases with confirmed (1 case showing constitutional mutation of *MLH1* gene) or highly suspected (6 cases) Lynch syndrome (see below).

**Table 1:** Clinicopathologic and prognostic features of the 66 stage II small bowel adenocarcinomas

	*N* of cases (%)	*N* of deaths (%)	HR (95% CI), *P* value (Cox)
Age at SBA diagnosis			
> 62 years	33 (50)	12 (36)	2.89 (0.93–8.99), *P* = 0.050
< 62 years	33 (50)	4 (12)	1
Sex			
Male	42 (64)	11 (26)	1.87 (0.64–5.45), *P* = 0.237
Female	24 (36)	5 (21)	1
Site			
Duodenum	5 (8)	2 (40)	2.07 (0.47–9.17), *P *= 0.381
Jejunum/ileum	61 (92)	14 (23)	1
Predisposing condition			*P *= 0.056
Crohn’s disease	20 (30)	6 (30)	6.91 (0.83–57.45), *P *= 0.074
Lynch syndrome^a^	7 (11)	2 (29)	6.25 (0.57–68.98), *P *= 0.135
None (sporadic)	18 (27)	7 (39)	9.34 (1.15–76.04), *P *= 0.037
Celiac disease	21 (32)	1 (5)	1
Celiac disease			
Yes	21 (32)	1 (5)	0.13 (0.02–0.98), *P *= 0.008
No	45 (68)	15 (33)	1
T stage			
T4	17 (26)	7 (41)	2.60 (0.97–7), *P *= 0.068
T3	49 (74)	9 (18)	1
R status			
R1	6 (9)	2 (33)	2.46 (0.55–10.96), *P* = 0.291
R0	60 (91)	14 (23)	1
Number of LN examined			
Low	29 (44)	10 (34)	2.03 (0.73–5.63), *P *= 0.166
Adequate	37 (56)	6 (16)	1
High-risk features, any			
Yes	41 (62)	14 (34)	3.73 (0.84–16.57). *P* = 0.083
No	25 (38)	2 (8)	1
Vascular or perineural invasion			
Yes	35 (53)	9 (26)	1.23 (0.46–3.31), *P* = 0.681
No	31 (47)	7 (23)	1
Histologic grade			
High (G3)	23 (35)	7 (30)	1.53 (0.57–4.13), *P *= 0.403
Low (G1–G2)	43 (65)	9 (21)	1
Extended high-risk features			
Yes	52 (79)	15 (29)	3.33 (0.44–25.43), *P* = 0.166
No	14 (21)	1 (7)	1
Histologic subtype group			
Cohesive (glandular/medullary)	54 (82)	9 (17)	0.23 (0.08–0.61), *P *= 0.006
Non-cohesive (diffuse/mixed)	12 (18)	7 (58)	1
MMR-d			
Yes	28 (42)	3 (11)	0.25 (0.07–0.87), *P *= 0.014
No	38 (58)	13 (34)	1

*CI* confidence interval; *HR* hazard ratio; *LN* lymph nodes, *MMR*-*d* mismatch repair deficiency; *SBA* small bowel adenocarcinoma

^a^Including 1 genetically confirmed Lynch syndrome patient and 6 cases strongly suspected for Lynch syndrome due to their histomolecular profiles

**Fig. 1:**
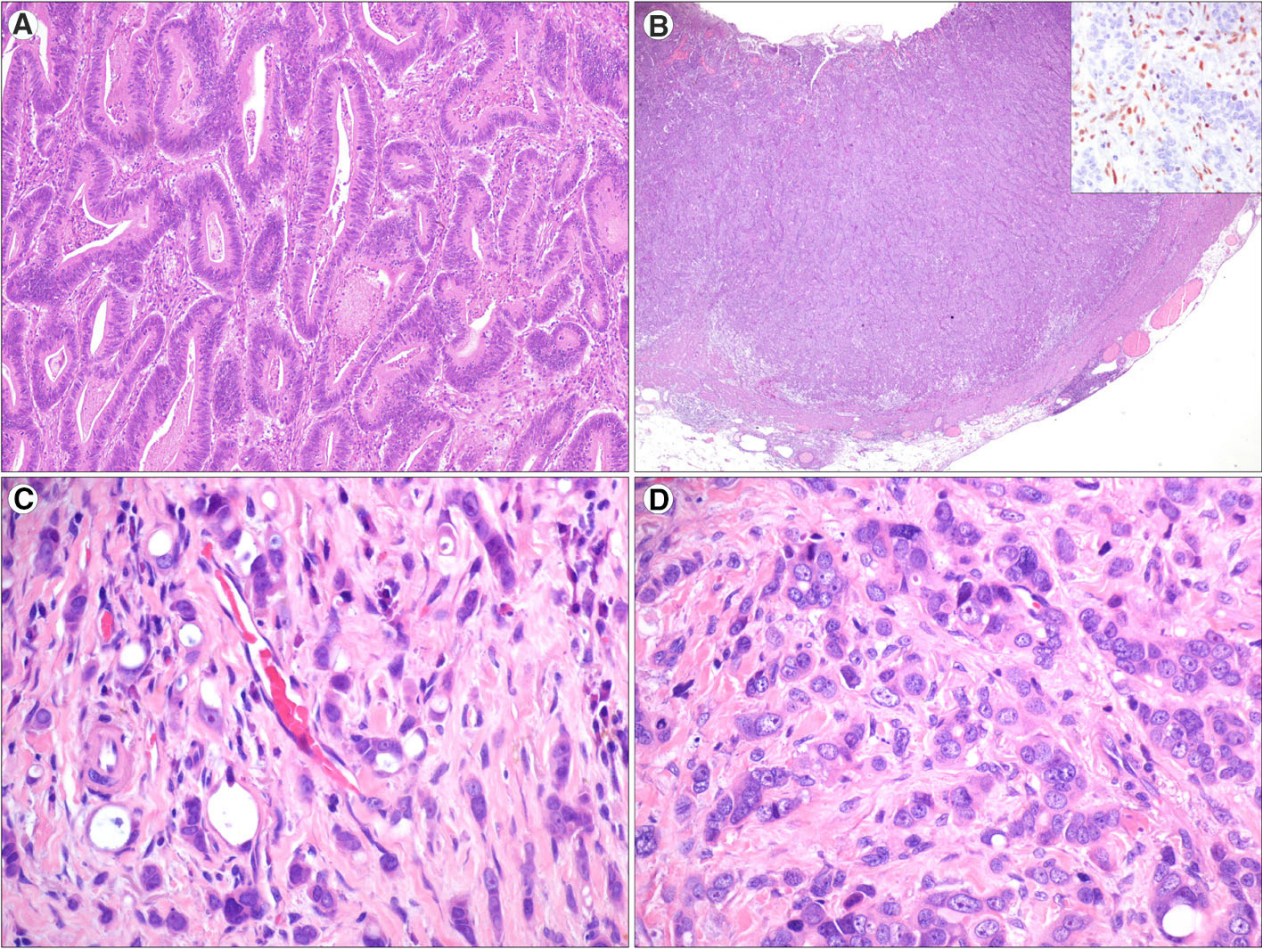
Small bowel adenocarcinoma (SBA) histologic subtypes (hematoxylin and eosin). **a** A glandular-type SBA, exhibiting well-formed glandular structures (original magnification × 200). **b** A medullary-type SBA, showing a solid pattern and a pushing border. As seen in the *inset* (MLH1 immunohistochemistry), tumor cells lacked nuclear expression of MLH1, which was retained in stromal and inflammatory cells (original magnification × 20, original magnification of *inset* × 200). **c** A mixed-type SBA, showing a combination of both glandular structures (on the *left*) and poorly cohesive cells (on the *right*), dispersed in a desmoplastic stroma (original magnification × 200). **d** A diffuse-type SBA, characterized by poorly cohesive, atypical cells in a desmoplastic stroma (original magnification × 200)

A fraction of such cases entered previous studies from the Small Bowel Cancer Italian Consortium.^10^^,^^12^^,^^20^^,^^25^^,^^26^ Patients were predominantly males (64%), the median age at SBA diagnosis was 61.5 years, and the tumor was mainly located in the jejunum-ileum (92%). Histologically, SBAs were classified as glandular (74%), medullary (8%), mixed (9%), or diffuse/poorly cohesive (9%) (Fig. 1). In 44% of cases, the total number of examined lymph nodes was under the cut-off endorsed by the NCCN guidelines. The median number of lymph nodes harvested was eight. At least one high-risk or extended high-risk feature was present in the majority of cases (62% and 79%, respectively).

MMR-d, confirmed by molecularly assessed MSI status in all cases, was found in a high percentage (42%) of stage II SBAs. Among the 28 cases showing an MMR-d phenotype, 25 had a combined loss of MLH1 and PMS2 (including 19 SBAs with *MLH1* methylation and 6 without *MLH1* methylation, one of which was in a confirmed Lynch syndrome patient). MLH1 methylated cases comprised 16 SBAs associated with celiac disease, 2 sporadic SBAs and 1 Crohn’s disease-related SBA. One SBA arising in a Crohn’s disease patient showed a combined loss of MSH2 and MSH6 (without known germline MMR gene mutations) and 2 SBAs featured an isolated loss of MSH6, one of which occurred in a Crohn’s disease patient without constitutional MMR gene mutations. The other patient with isolated MSH6 loss, and the 5 cases with combined MLH1/PMS2 loss in the absence of *MLH1* gene hypermethylation (both histo-molecular patterns highly suggestive of Lynch syndrome^19^), were classified as highly suspected Lynch syndrome; unfortunately, germline tests confirming constitutional MMR gene mutation were not available for these 6 patients.

Only a minority of patients (5 cases, 8%, median age at diagnosis 47 years, including 3 males and 2 females), underwent ACT (FOLFOX regimen, 6 months). Four of these 5 patients were affected by celiac disease (2 cases) or Crohn’s disease (2 cases), while the remaining patient had a sporadic SBA. Four of such SBAs were located in the jejunum-ileum and the other one in the duodenum. Two of the 5 SBAs harbored MMR-d (both in celiac patients), and all five cases exhibited at least one high-risk feature with a low lymph node count present in 4 out of 5 cases.

**Fig. 2:**
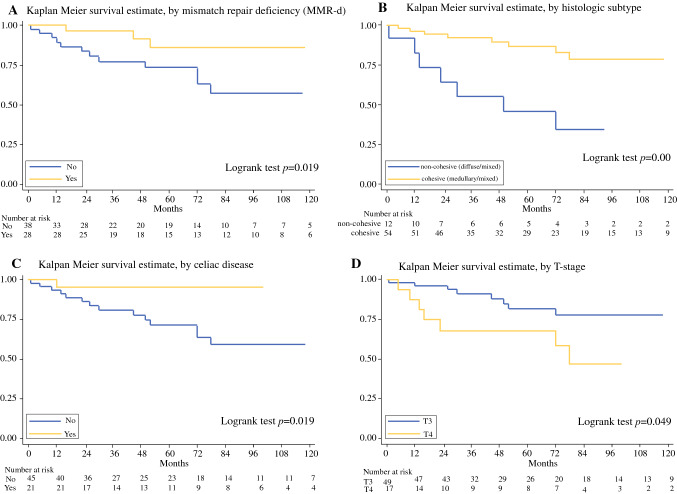
Kaplan-Meier cancer-specific survival estimates by MMR status (**a**), histologic subtype (**b**), celiac disease etiology (**c**) and T stage (**d**)

Patients were followed up for a median of 73 months (25–75th percentile: 35–118). Cancer-specific survival analysis identified MMR-d as a significant predictor of favorable survival (HR 0.25, 95% CI 0.07–0.87, Table 1 and Fig. 2a). MMR status was not significantly correlated with a series of other parameters potentially affecting its prognostic value, with the only exceptions being the underlying predisposing clinical condition (notably, most MMR-d cases arose in celiac disease patients), R status, and histologic subtype (Table 2). Celiac disease (*P* = 0.011) and histologic subtype (*P* = 0.031) also proved to be significantly associated with MMR-d in a multivariate exact logistic regression model.

**Table 2:** Relationship between mismatch repair status and clinicopathologic features in the 66 stage II small bowel adenocarcinomas

	MMR-d	MMR-p	*P* value
Number of cases (%)	28 (42)	38 (58)	
Age at SBA diagnosis, years, mean ± SD	60.1 ± 16.7	61.7 ± 12.9	0.659
Sex, *N* (%)			0.797
Female	11 (39)	13 (34)	
Male	17 (61)	25 (66)	
Predisposing condition, *N* (%)			< 0.001
Celiac disease	16 (57)	5 (13)	
Crohn’s disease	3 (11)	17 (45)	
Lynch syndrome^a^	7 (25)	0 (0)	
None (sporadic)	2 (7)	16 (42)	
Site, *N* (%)			1.000
Duodenum	2 (7)	3 (8)	
Jejunum/ileum	26 (93)	35 (92)	
T level, *N* (%)			0.576
T3	22 (79)	27 (71)	
T4	6 (21)	11 (29)	
R status, *N* (%)			0.035
R0	28 (100)	32 (84)	
R1	0 (0)	6 (16)	
Adequate number of LN, *N* (%)			1.000
Yes	16 (57)	21 (55)	
No	12 (43)	17 (45)	
Any high-risk features, *N* (%)			0.609
No	12 (43)	13 (34)	
Yes	16 (57)	25 (66)	
Vascular or perineural invasion, *N* (%)			0.456
No	15 (54)	16 (42)	
Yes	13 (46)	22 (58)	
Histologic grade, *N* (%)			0.300
Low (G1–G2)	16 (57)	27 (71)	
High (G3)	12 (43)	11 (29)	
Any extended high-risk features, *N* (%)			0.555
No	7 (25)	7 (18)	
Yes	21 (75)	31 (82)	
Histologic subtype, *N* (%)			< 0.001
Medullary	5 (18)	0 (0)	
Glandular	23 (82)	26 (68)	
Diffuse	0 (0)	6 (16)	
Mixed	0 (0)	6 (16)	
Histologic subtype group, *N* (%)			0.001
Cohesive	28 (100)	26 (68)	
Non-cohesive	0	12 (32)	

*LN* lymph nodes; *MMR*-*d* mismatch repair deficient; *MMR*-*p* mismatch repair proficient; *SBA* small bowel adenocarcinoma; *SD* standard deviation

^a^Including 1 genetically confirmed Lynch syndrome patient and 6 cases strongly suspected for Lynch syndrome due to their histomolecular profiles

In particular, all medullary-type cancers were MMR-d whereas all mixed and diffuse cancers were MMR-p. Interestingly, histologic classification by itself was associated with patient outcome (Fig. 2b). Indeed, patients with glandular or medullary (cohesive) cancers showed a more favorable prognosis compared with those with a non-cohesive mixed-to-diffuse SBA (HR: 0.23, 95% CI 0.08–0.61, Table 1). In addition, a reduced risk of death was observed in celiac disease patients compared with the non-celiac ones (Fig. 2c), and in particular compared with patients with sporadic cancer (Table 1).

Among high-risk features (T4, R1 and low number of lymph nodes) only T4 versus T3 showed a significant association with a worse patient outcome (Fig. 2d), while R1 and a low number of lymph nodes examined revealed a non-significant trend toward decreased survival (Table 1). No significant difference was found between cases with or without lymphovascular/perineural invasion or between those of low (G1–G2) and high (G3) AJCC grade. Subsequent CD31 immunostaining did not add further cases with lymphovascular invasion to those detected in hematoxylin and eosin-stained tumor sections.

When the analysis was restricted to the MMR-p cases, only T4 retained its prognostic power (HR: 4.18, 95% CI: 1.10–15.88, *P* = 0.036), while the other parameters showed non-significant association with survival. In the MMR-d subset, no factor was associated with patient survival. Although pT stage lost its significance in a bivariable model adjusted for MMR-d status, it remained a significant predictor of patient outcome in bivariable models adjusted for etiology and histologic subtype (Table 3). MMR-d status, celiac disease and histologic subtype retained significance as prognostic markers in bivariable models adjusted for pT stage. Histologic subtype (cohesive versus non-cohesive) was also a significant prognostic parameter in a bivariable model adjusted for celiac etiology.

**Table 3:** Cancer-specific survival by bivariable Cox models of the 66 stage II small bowel adenocarcinomas

Bivariable model	HR (95% CI)	*P* value (Cox)
*Model#1*		0.009
MMR-d		
Yes	0.24 (0.07–0.87)	0.03
No	1	
T stage		
T3	1	
T4	2.63 (0.97–7.11)	0.058
*Model#2*		0.004
Celiac disease		
Yes	0.12 (0.02–0.94)	0.043
No	1	
T stage		
T3	1	
T4	2.76 (1.02–7.45)	0.045
*Model#3*		0.001
Histologic subtype		
Cohesive	0.15 (0.05–0.45)	0.001
Non-cohesive	1	
T stage		
T3	1	
T4	4.16 (1.41–12.26)	0.01
*Model#4*		0.003
Celiac disease		
Yes	0.18 (0.02–1.46)	0.109
No	1	
Histologic subtype		
Cohesive	0.33 (0.12–0.90)	0.031
Non-cohesive	1	

*CI* confidence interval; *MMR-d* mismatch repair deficiency

## Discussion

In this study, we found that MMR-d/MSI phenotype and glandular/medullary (i.e., cohesive) histologic subtype were associated with a more favorable cancer-specific survival in patients with resected stage II SBAs, whereas T4 correlated with a worse prognosis.

MMR-d, which leads to the MSI phenotype and is associated with high lymphoid response in solid tumors, has been associated with better survival in resected SBAs.^12^^–^14 However, its prognostic value in stage-inclusive multivariate models was unclear. To the best of our knowledge, this is the first study that found a significant association of MMR-d and cancer-specific survival in stage II SBAs. This finding supports the NCCN guidelines which do not indicate ACT for patients with MMR-d stage II resected SBAs.

From our findings it appears that stage II SBAs are enriched with MMR-d cancers and especially with those characterized by MLH1/PMS2 loss. Interestingly, González et al. also found a higher percentage (26%) of MMR-d in stage II SBAs compared with stage III (18%) or stage IV (0%) SBAs.^13^ In addition, fewer MMR-d/MSI-high cancers were found among stage IV colorectal cancers.^27^ This behavior might be explained in part by the more prominent anti-tumor immune response which is frequent and well documented in MMR-d cancers. Furthermore, it should be pointed out that in our series, most MMR-d stage II SBAs were celiac disease-associated and the *MLH1* gene was hypermethylated, with consequent loss of immunohistochemical expression of the MLH1 protein. We also confirmed in the present series of stage II SBAs that celiac disease patients show better prognosis compared with the remaining SBA cases, as previously reported by our group.^10^^,^^12^^,^^20^^,^^28^ Notably, the high predominance of MMR-d among celiac disease-associated SBAs (76% in the present study) has already been reported.^11^

Tumor stage is a strong prognostic factor in SBAs.^29^ An important issue in staging gastrointestinal tumors, including SBAs, is the number of lymph nodes which need to be examined for an accurate tumor staging. The lower the number of lymph nodes harvested, the higher is the risk of downstaging. Among patients with stage II SBAs, 5-year cancer-specific survival has been found to be strongly associated with the total lymph nodes assessed, ranging from 44% when no lymph nodes were evaluated to 83% when more than 7 lymph nodes were analyzed.^29^ In a large Surveillance, Epidemiology and End Results (SEER) database study, harvesting at least 9 and 5 lymph nodes for jejuno-ileal and duodenal SBAs, respectively, resulted in the greatest prognostic difference, and a recent propensity score-adjusted analysis indicated increased overall and cancer-specific survival in patients with the retrieval of at least 9 lymph nodes.^30^^,^^31^ On these bases, NCCN guidelines recommend retrieving a minimum of 8 lymph nodes for all SBAs. In our study, we found that a lower number of lymph nodes examined, found in 44% of our cases, was associated with a non-significant trend towards a worse outcome in stage II SBAs. A possible reason for the absence of statistical significance may be the limited sample size.

T4 stage, resection margin involvement, vascular or perineural invasion, and duodenal site have been reported as adverse prognostic factors in SBAs.^3^^,^^24^^,^^29^^,^^32^ We proved that T4 represents an adverse prognostic factor in stage II SBAs, while we found only a non-significant trend towards a less favorable outcome for resection margin involvement, lymphovascular/perineural invasion, and duodenal location. Although T stage lost its significance in a bivariable model adjusted for MMR-d, T4 was a significant negative predictor of outcome in the MMR-p subset.

Tumor differentiation grade according to AJCC criteria, based on the proportion of tumor composed by glands, was not significantly associated with survival in our series, which is at variance with the findings by Overman et al.^29^ A possible reason for this discrepancy may be the relative abundance in our series of medullary-type cancers, which are, by definition, poorly differentiated morphologically, despite their generally favorable prognosis. Indeed, we found that a histologic classification, whereby glandular/medullary cohesive cancers were separated from poorly cohesive diffuse-to-mixed cancers, was highly associated with prognosis, the former showing much longer survival than the latter. We argue that such a diffuse/mixed versus cohesive histologic classification might be incorporated as a feature for selecting SBA patients for ACT.

The role of ACT in SBAs is controversial, especially for stage II disease. In a meta-analysis of 15 studies, no significant effect of ACT on survival of SBA patients was found.^33^ However, a recent study showed that ACT was associated with improved overall survival in patients with stage II–IV SBA in a multivariate analysis stratified by stage.^34^ An international phase III trial (Prodige 33-BALLAD, NCT02502370), investigating the potential benefits of ACT in stage I–III SBAs, is still ongoing.^35^^,^^36^

In conclusion, because of their proved prognostic impact in stage II disease, MMR (or MSI) status and histotype may help identify patients with stage II SBAs who may benefit more from ACT. Among those with MMR-p SBAs, T4 tumors may require more aggressive therapeutic strategies.
